# Ignored or underestimated - evaluation and treatment of cardiovascular risk factors in patients with adrenal insufficiency

**DOI:** 10.1007/s12020-025-04192-0

**Published:** 2025-02-25

**Authors:** Anja Wasmuth, Iris van de Loo, Julia Domberg, Birgit Harbeck

**Affiliations:** 1 III. Department of Medicine, University Medical Center Hamburg-Eppendorf, Hamburg, Germany; 2 Practice for Internal medicine, Diabetology und Endocrinology Bremen, Bremen, Germany; 3 Practice for Internal medicine, Diabetology und Endocrinology “Am alten Handelshafen”, Leer, Germany; 4 MVZ Amedes Experts, Endocrinology, Hamburg, Germany

**Keywords:** Adrenal insufficiency, Cardiovascular risk, Cardiovascular risk assessment, Quality of healthcare

## Abstract

**Purpose:**

Patients with adrenal insufficiency (AI) are known to have a higher cardiovascular risk (CVR) than the normal population. In particular arteriosclerosis, coronary heart disease, arterial hypertension, hyperlipoproteinemia as well as metabolic disturbances contribute to the increased morbidity and mortality. Aim of this study was to evaluate known CVR factors along with the quality of care by the treating physicians.

**Methods:**

To this end the medical records of AI patients were screened for CVR factors and the treatment initiated was documented. In addition, a questionnaire evaluating CVR factors was analyzed if available.

**Results:**

In total, 327 AI patients were included in the study. At least 298 of these patients were found to have one or more CVR factors. Ninety-one patients were diagnosed with arterial hypertension, of these 40 patients (44%) still showed increased blood pressure (BP) values. Of all AI patients, about 25% (*n* = 83) did not have measurements to calculate their BMI, even though obesity is known as a major risk factor for cardiovascular events. Out of 46 patients with diabetes, one-quarter still had increased HbA1c values. Regarding hyperlipoproteinemia, only 2% of AI patients achieved normal lipid values across all parameters (*n* = 8). Interestingly, at least one lipid variable was untested in 150 patients (46%).

**Conclusion:**

Our study demonstrates (1) the high rate of CVR factors in AI patients, leading to increased morbidity and eventually mortality, (2) AI patients are inadequately monitored and treated for CVR factors, (3) treating physicians should be aware of this risk to minimize complications where possible.

## Introduction

**Fig. 1:**
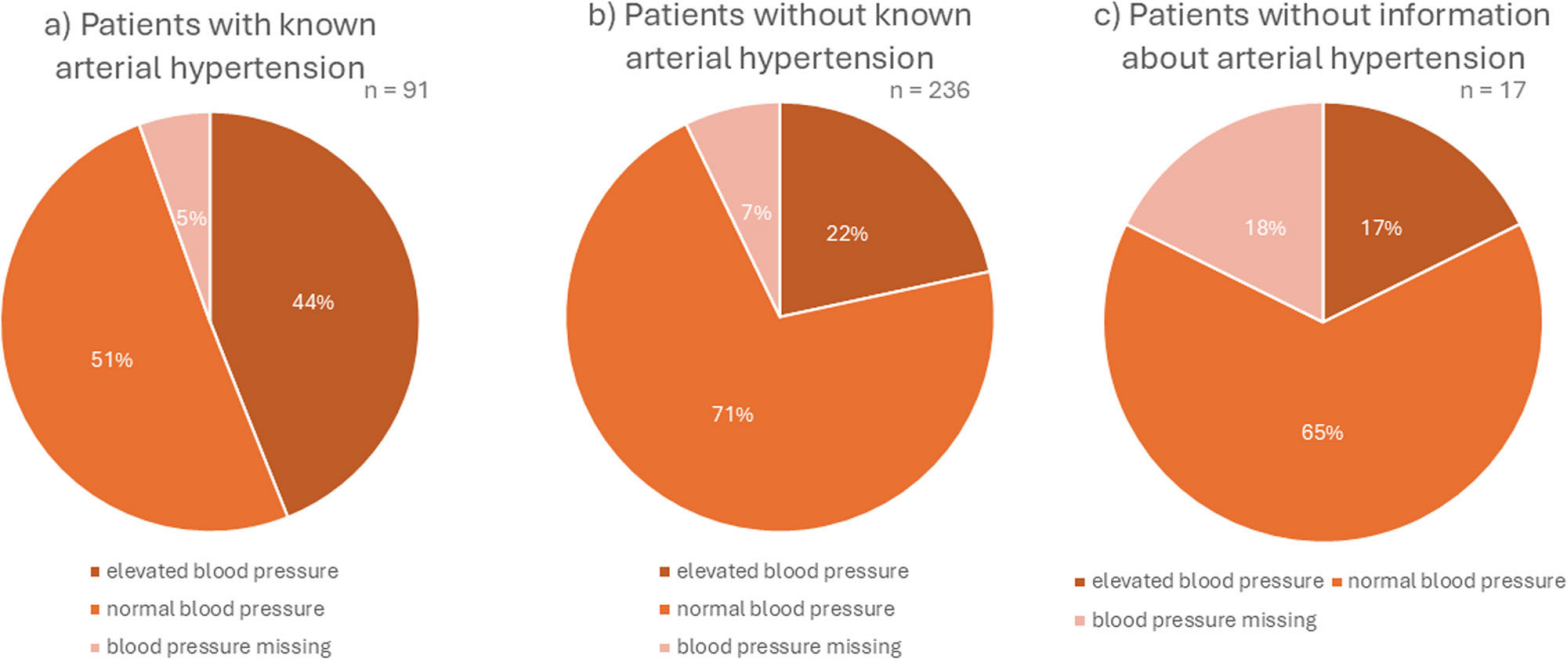
Arterial hypertension and blood pressure. **a** Patients with known arterial hypertension, **b** patients without known arterial hypertension, **c** patients without information about arterial hypertension

**Fig. 2:**
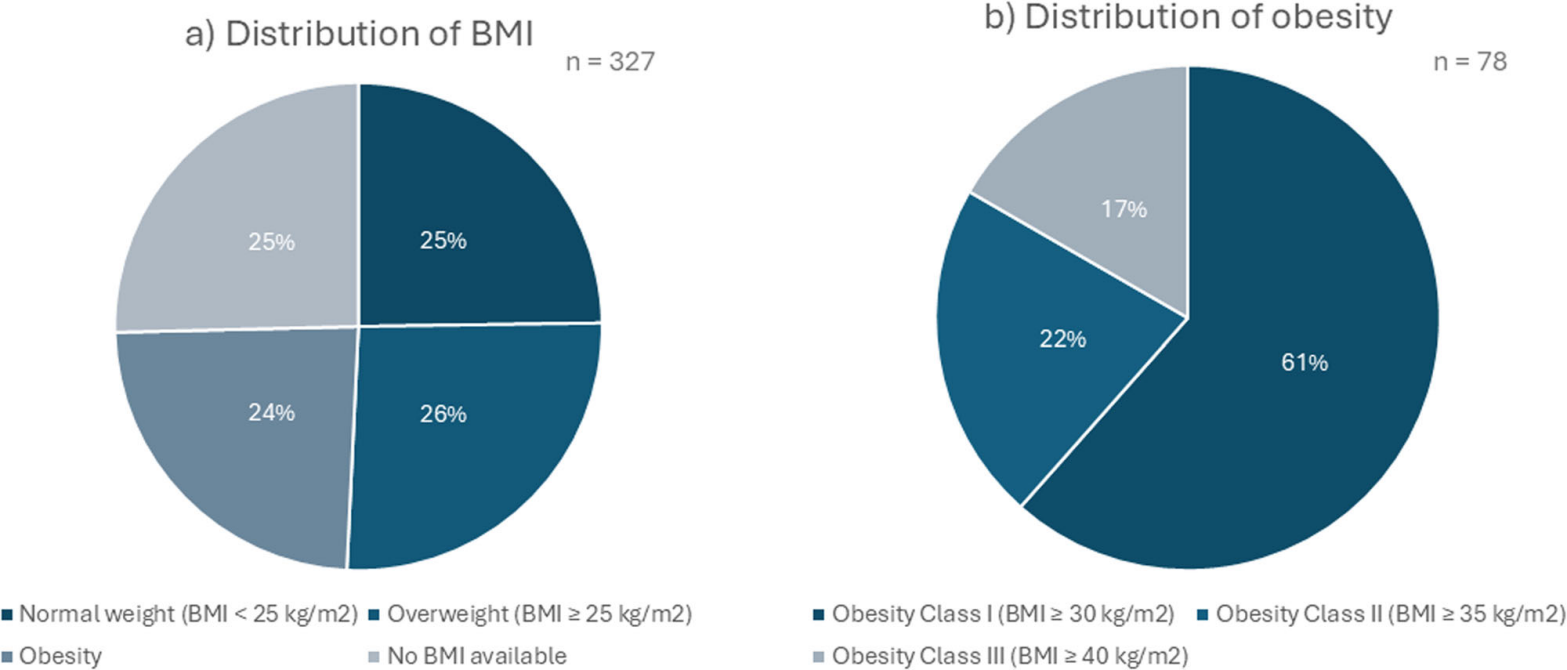
Overview of BMI distribution in AI patients. **a** distribution of BMI, **b** distribution of obesity classes in AI patients with obesity

**Fig. 3:**
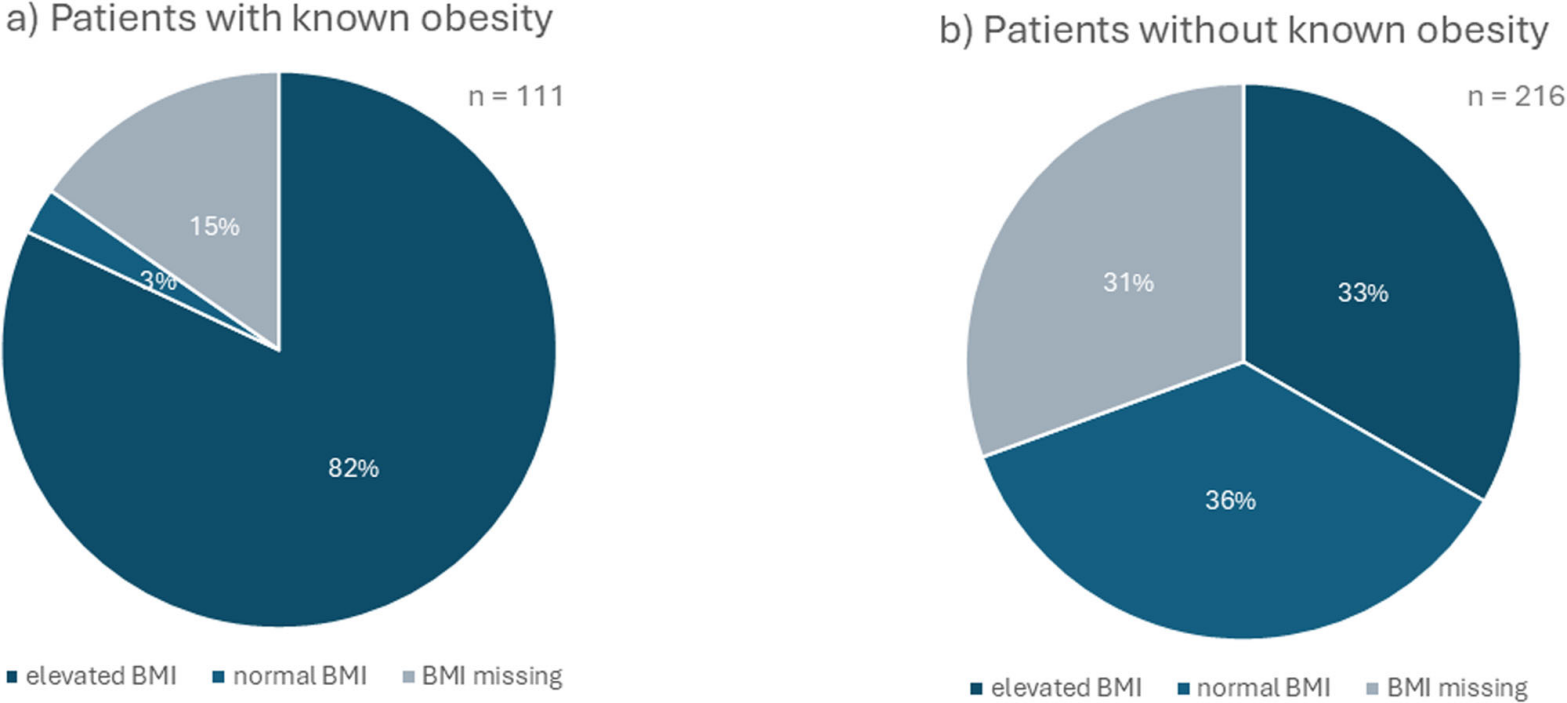
Obesity and BMI. **a** with diagnosis of obesity, **b** patients without diagnosis of obesity

**Fig. 4:**
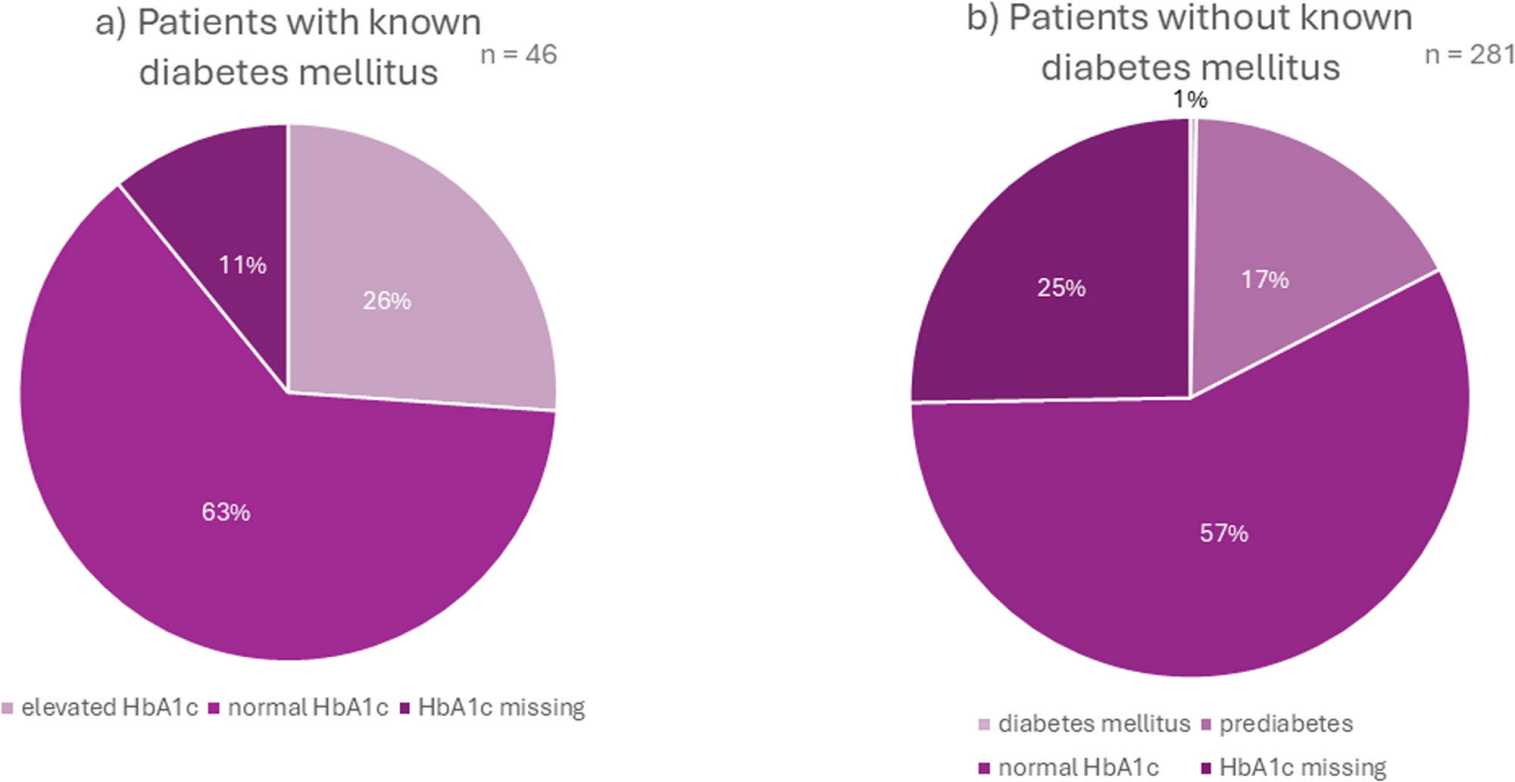
Diabetes mellitus and HbA1c levels. **a** patients with known diabetes mellitus, **b** patients without known diabetes mellitus

**Fig. 5:**
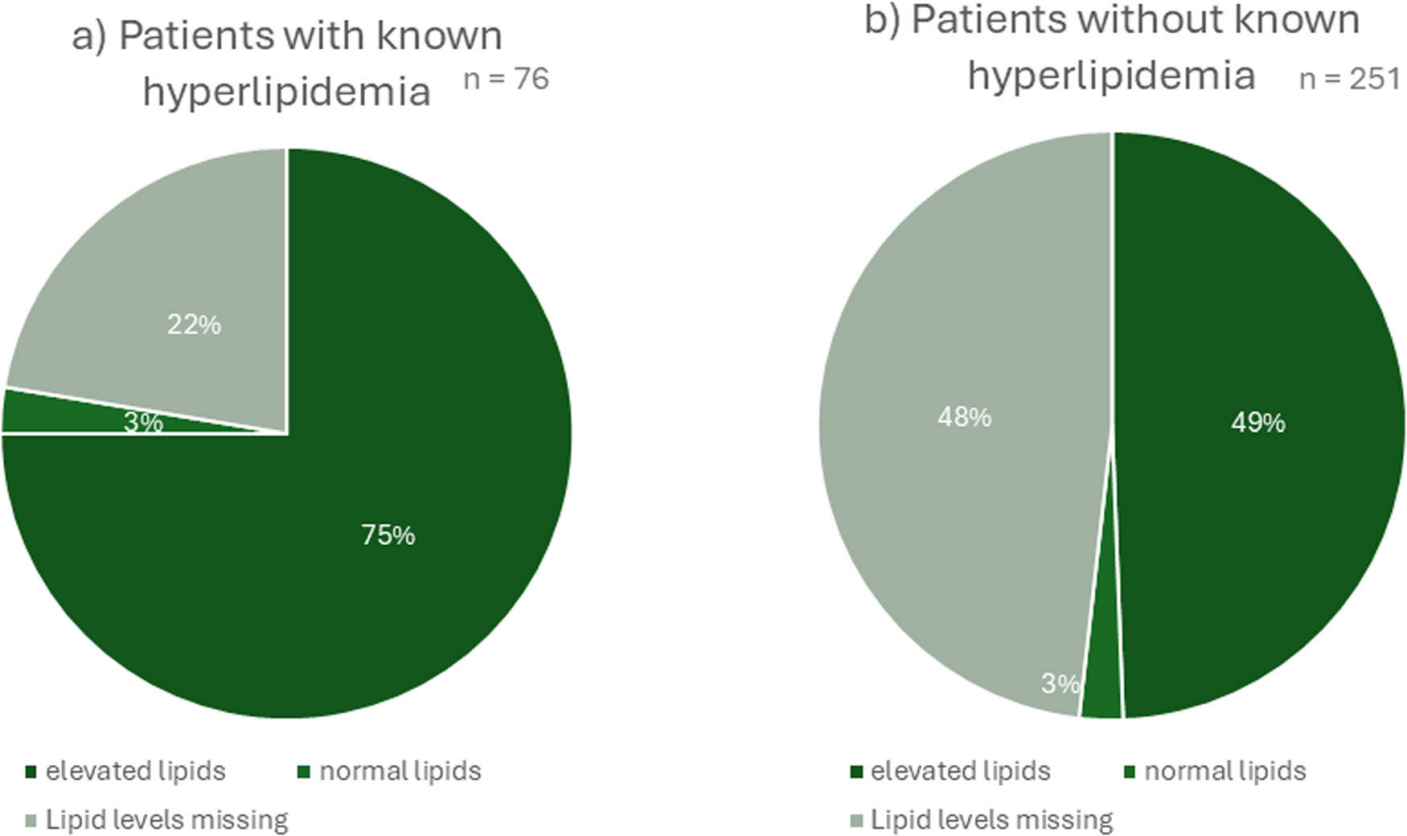
Hyperlipidemia and lipid levels. **a** patients with known hyperlipidemia, **b** patients without known hyperlipidemia

**Fig. 6:**
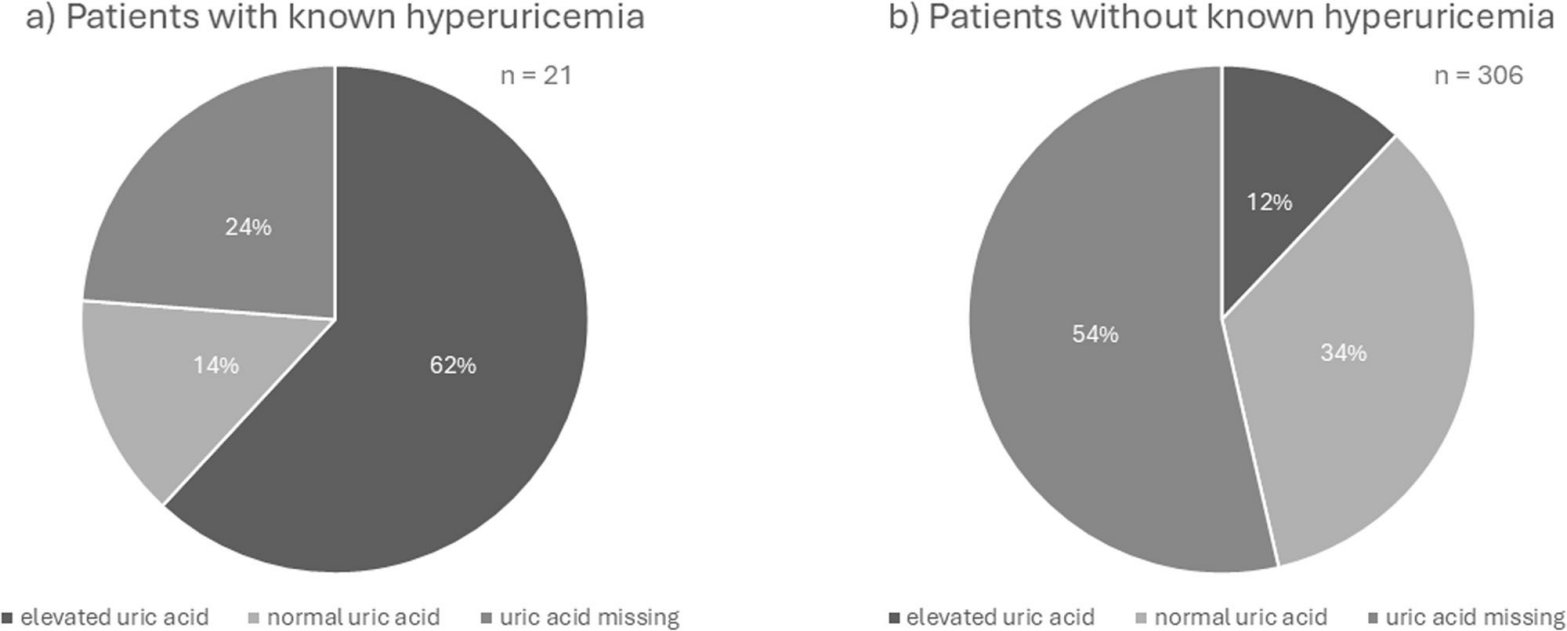
Hyperuricemia and uric acid levels. **a** patients with known hyperuricemia, **b** patients without known hyperuricemia

Adrenal insufficiency (AI) is a rare endocrine disorder affecting 100–126 individuals per million for primary adrenal insufficiency (PAI) [1] and about 400 individuals per million for secondary adrenal insufficiency (SAI) [2]. The pathophysiological mechanism is the inability of the adrenal glands to achieve adequate hormonal production due to adrenal disease (PAI) or due to malfunction of the pituitary gland (SAI). Tertiary adrenal insufficiency (TAI) comprises the suppression of the hypothalamic-pituitary-adrenal axis by exogenous glucocorticoid therapy. All types of AI lead to a lack of endogenous glucocorticoids (GC) that must be substituted to prevent patients from harmful symptoms or even death. Therefore, lifelong glucocorticoid replacement is essential in the treatment of PAI and SAI. The most frequently used GC formula for replacement therapy is hydrocortisone (HC), which has also been modified in the last years [3, 4]. Conventional glucocorticoid replacement therapy aims to mimic the physiological circadian rhythm of cortisol secretion as closely as possible to avoid side effects due to phases of hypo- and hypercortisolism. Usually, the administration of hydrocortisone (15–25 mg/d), is divided into two or three single doses [5, 6]. However, this substitution regimen leads to unphysiological plasma cortisol levels. These imbalances may foster a higher prevalence of coronary heart disease, and major adverse coronary events, and lead to increased mortality. While increased levels of GC are associated with obesity, high blood pressure, and hyperglycemia, decreased blood levels of cortisol result in a compensatory secretion of inflammatory mediators such as Interleukin-1 (IL-1), Interleukin-6 (IL-6) and tumor necrosis factor (TNF) [7, 8] Figs. 1–6.

Patients with AI are known to have a higher CVR (cardiovascular risk) than the normal population [7, 9]. In particular, arteriosclerosis, coronary heart disease, arterial hypertension, hyperlipoproteinemia as well as metabolic disturbances contribute to increased morbidity and mortality [9–13]. Hydrocortisone doses above 20 mg per day seem to be associated with increased CVR due to the higher prevalence of common metabolic risk factors [6]. Thus, GC therapy may play a pivotal role in some of the well-known risk factors for cardiovascular disease (CVD). A similar observation was done in a population-based case-control study with 50,656 patients having received at least one prescription for systemic or non-systemic GCs [14] who showed an increased risk for cardiovascular and cerebrovascular events when compared to matched controls without GC intake with an adjusted odds ratio of 1.25 (95% confidence interval (CI) 1.21–1.29) [14]. Likewise, an increased cerebrovascular risk was found in GC-treated patients with SAI [15].

Aim of the study was to evaluate known CVR factors in AI patients as well as the quality of care by the treating physicians.

## Materials and methods

In this retrospective study, we screened the medical records of AI patients in three different endocrine outpatient practices (Bremen, Hamburg, and Leer) for CVR factors and documented the treatment initiated. If available, a questionnaire evaluating CVR factors was additionally analyzed. The questionnaire asked for existing diseases associated with increased CVR and family history concerning these diseases, as well as data for adjustable risk factors, such as BP (blood pressure) or weight and height. Statistical analysis was performed by IBM SPSS Statistics, Version: 29.0.0.0 (241). We completed an ANOVA Analysis to compare the three participating outpatient practices in terms of the availability of data. A *p*-value less than 0.05 was considered significant.

Patients with TAI or with an unknown cause were excluded. Following inclusion criteria were applied: patients over 18 years of age, PAI or SAI, written informed consent for the questionnaire.

Blood parameters (lipids [total cholesterol, LDL cholesterol, HDL cholesterol, triglycerides, and Lp(a)], uric acid, HbA1c) were analyzed using standardized measuring techniques. Lipid values were taken in a fasting state. The reference values can be found in Appendix table 1.

Blood pressure was either a self-measurement at home or measured in the doctor’s office. Values for weight and height were obtained in the same way.

Questionnaire see appendix.

## Results

In total, 363 AI patients were found, and 327 of these fulfilled the inclusion criteria (149 PAI [46%], 178 SAI [54%]). We included 216 women and 111 men, with most of the patients being between 31 and 50 years (33%) and between 51 and 69 years (32%). Twenty-one percent were 70 years and older. Mean age was 53 years (range 18–94). The vast majority of patients (*n* = 264) were on conventional hydrocortisone, 36 patients were treated with modified released hydrocortisone (Plenadren®) and 8 patients took prednisolone. However, 19 participants were substituted with a fixed combination of conventional hydrocortisone, modified released hydrocortisone resp. prednisolone. Interestingly, dexamethasone was given additionally to Plenadren® or prednisolone in two of these cases.

Mean dosage of GC (dosing equivalents relative to hydrocortisone) was 20.1 mg with a standard deviation of 9.9 mg. Two-hundred-thirty-six patients took less than 30 mg (79%), whereas 48 AI patients took 30 to 35 mg (15%) and 21 participants took even more than 35 mg (6%). Ninety-two PAI patients were taking fludrocortisone (62%) with a mean dose of 0,09 mg.

Of the 327 included patients, 299 were found to have one or more CVR factors. However, in 28 patients the risk factors were not sufficiently assessed, meaning they did not have any data for one or more risk factors. We found the following CVR factors: arterial hypertension, arteriosclerosis, coronary heart disease, peripheral artery disease, stroke, diabetes mellitus, hyperlipoproteinemia, hyperuricemia, obesity, sleep apnea syndrome, coagulation disorder, aneurysm, smoking, positive family history for the conditions mentioned above, as well as elevated values of lipids, uric acid, HbA1c, BMI or blood pressure. In total we identified 27 risk factors.

### Risk factors

Only one patient did not have any risk factors. Most AI patients (*n* = 299) were found to have at least one risk factor (91%).

Between the three participating locations, there were significant differences regarding the evaluation of CVR factors.

On average, the patients in Hamburg were found to have about ten missing risk factors, whereas in Bremen about two factors were missing, and in Leer approximately three. There was a significant difference between Hamburg and Leer (*p* < 0.001) and Hamburg and Bremen (*p* < 0.001).

### Arterial hypertension

Elevated blood pressure (BP) was the leading global contributor to premature death in 2015 and is above all related to cardiovascular events such as stroke, myocardial infarction, heart failure, peripheral artery disease, and end-stage renal disease [16, 17]. Hypertension is defined as office systolic BP values > 140 mmHg and/or diastolic BP values > 90 mmHg [18].

In 91 patients (27.8%) arterial hypertension was documented with 44% (*n* = 40) of them still having a higher BP in one measurement. Almost half of the AI patients with known hypertension showed normal or low BP (*n* = 46) and five patients had no documented BP (5.5%) at all (Fig. 1).

In patients, classified as not having arterial hypertension (*n* = 236), interestingly 21.6% showed elevated BP (*n* = 51). For 168 patients normal or low BP was documented (71.2%). Similar to patients with known arterial hypertension, a relevant proportion of this patient group (7.2%) did not provide any BP value (*n* = 17).

Concerning medication, three-quarters of the AI patients with known hypertension did not take any antihypertensive drugs according to the medical records. Furthermore, in patients who currently demonstrated high BP measurements, there were 65.9% who did not take any antihypertensive medication. Antihypertensive dose adjusting was performed by the treating family physicians in almost all cases.

### Obesity

Overweight and obesity are defined as abnormal or excessive fat accumulation that may impair health. Obesity is a body mass index (BMI) greater than or equal to 30 kg/m^2^ [19]. A BMI between 25 kg/m^2^ and 29,9 kg/m^2^ is considered overweight. Typical complications comprise type 2 diabetes, hypertension, fatty liver disease, and obstructive sleep apnea [20]. The distribution of BMI and obesity is shown in Fig. 2.

In total, 163 patients were overweight or obese. The threshold for obesity was reached by 78 of the AI patients with 13 having a BMI of 40 or higher.

About 80% of all AI patients with a documented diagnosis of obesity in the medical records had an elevated BMI (>25 kg/m^2^, *n* = 91), but only 60.3% were in fact obese (BMI > 30 kg/m^2^). However, 17 of these cases (15.3%) had no documented BMI at all. Three of the patients with documented diagnosis of obesity had BMI values in the normal range (almost 3%) (Fig. 3).

Surprisingly, 5% of all AI patients who were not classified as suffering from obesity, were in fact obese (*n* = 11). Only 36.1% of this patient group had BMI values in the normal range (*n* = 78). The threshold for overweight was reached by 72 patients (33.3%). In 66 cases weight measurements were missing (30.6%). No weight measurements were documented for 66 patients (30.6%).

Patients with elevated BMI were often found to have more than one CVR factor. Merely five of these patients had elevated BMI as the only risk factor. Fifty-three of these patients were also known to have arterial hypertension (33%). Ninety-one patients with elevated BMI showed increased lipid levels (56%) as well.

Patients with obesity did not get any medical therapy to reduce weight except lifestyle modifications.

### Diabetes mellitus

The 48 mmol/mol (6.5%) HbA1c threshold was used to monitor blood glucose. Target HbA1c levels for patients with type 1 diabetes mellitus (T1DM) are between 6.5 and 7.5% [21]. The target levels for type 2 diabetes mellitus (T2DM) were recently defined individually between 6.5 and 8.5% [22]. For this study, we used the 7.5% threshold for both types. Typical complications of diabetes mellitus (DM) include cardiovascular, renal, peripheral vascular, ophthalmic, hepatic, or neurological diseases [23].

Although 46 patients suffered from DM, more than 1/4 (*n* = 12) still had increased HbA1c values (Fig. 4).

No DM was documented for 281 patients. However, in 17.5% of these cases, an elevated HbA1c was found (*n* = 49). The majority had prediabetes, only in one case the threshold for DM was reached.

Only four of the diabetic patients took antidiabetics. There were eleven patients with known DM still having HbA1c over 7.5% who did not take any antidiabetic medication. Interestingly, there were also four patients without known DM and no data for HbA1c who did take antidiabetic drugs.

### Hyperlipidemia

Hyperlipidemia is known to be an important CVR factor [24]. The local laboratory threshold for total cholesterol was 200 mg/dl. The most important subgroup is LDL cholesterol as it is the major risk factor in the formation of atherosclerotic plaques [24]. LDL cholesterol is clearly elevated if it is 155 mg/dl or higher. Triglycerides act as a predictive marker for cardiovascular events [25]. They are marked as elevated when over 150 mg/dl. The role of Lp(a) has become more important over the last few years; it is considered to be an independent risk factor for CVD [26, 27]. If Lp(a) is 72 nmol/l or higher it is regarded as elevated.

Concerning hyperlipidemia, about 55% of all AI patients had at least one elevated lipid level (*n* = 181). Only three patients demonstrated lipids in the normal range in each subgroup (total cholesterol, LDL cholesterol, HDL cholesterol, triglycerides, and Lp(a)). Hundred-fifty of all AI patients were not adequately assessed, meaning that at least one value was unavailable. However, the existing lipid levels were normal. Out of the 181 patients with at least one elevated lipid, only 57 had documented hyperlipidemia. Seventeen patients with known hyperlipidemia had not been screened for elevated lipids (22.4%) (Fig. 5).

Only 17% of AI patients had data for Lipoprotein a (Lp(a)). Surprisingly no AI patient in Leer and only nine AI patients in Bremen had data for Lp(a).

In total, 126 patients had elevated total cholesterol levels (38.5%). There were six patients with data for total cholesterol but no further evaluation of the subgroups. In addition to elevated cholesterol levels, 53 AI patients also exhibited high values for LDL cholesterol.

Surprisingly, only 18 patients with known hyperlipidemia took lipid-lowering agents (23.7%). Moreover, in patients with at least one elevated lipid level, only 17.1% were taking lipid-lowering agents (*n* = 31).

### Hyperuricemia

Hyperuricemia is closely related to CVD with higher levels of uric acid (UA) being a risk for CVD [28]. It is defined as an elevation of serum UA (>6 mg/dL in women and > 7 mg/dL in men) [29]. We used the 5.7 mg/dL threshold as this was the laboratory reference. The underlying mechanisms of the increase in CVR are still not completely understood and are discussed controversially [30].

Only 21 of the AI patients were known to suffer from hyperuricemia (6%). Of these patients, five did not have data for uric acid (24%) (Fig. 6).

In 306 AI patients, no hyperuricemia was known. Though, 37 of these patients demonstrated elevated UA (12.1%). A big proportion (53.6%) did not have any measurements for UA (*n* = 164).

Unexpectedly, no patient with known hyperuricemia took uricostatic drugs but seven of the patients who, according to the documentation, did not know about their hyperuricemia.

## Discussion

Present data confirm the high prevalence of CVR factors in AI patients. Currently used glucocorticoid-replacement therapy regimens fail to mimic the physiological rhythm of endogenous cortisol secretion leading to temporary hyper- and hypocortisolism [5]. The induction of classical metabolic risk factors for vascular disease by glucocorticoids (e.g. dyslipidemia, obesity) as well as the still unphysiological exposure under conventional glucocorticoid replacement therapy with subsequently increased IL-6 and the epinephrine deficiency itself may promote the increased CVR in AI [7].

Dual-release hydrocortisone preparations such as Plenadren® or Chronocort® have been introduced in recent years to mimic the circadian rhythm of cortisol more closely to prevent serious side effects [31]. Nevertheless, we could demonstrate that still a considerable proportion of AI patients had more than one glucocorticoid replacement medication including dexamethasone that increases CVR even more ([6, 32]). Moreover, 21% of patients were treated with GC doses of ≥30 mg (dosage equivalents), fostering CVR ([33]). Since 62% of patients with PAI took fludrocortisone, there may also be a link between the usage of fludrocortisone and a higher CVR [34].

Our study shows that there were patients with known adjustable CVR factors for whom the current status was not examined. In particular, endocrinologists need to have these CVR factors in mind or at least make sure the general practitioners focus on the correct adjustment of lipids, BP, blood glucose, and UA and try to reduce obesity in these patients.

Three different endocrine practices were included in this study. Although specialized in endocrinology a remarkable gap was found among them when analyzing the assessment of CVR factors. In contrast to Hamburg, the practices in Bremen and Leer additionally treated the patients in a general approach. This could explain the higher occurrence of not adequately assessed risk factors of patients in Hamburg.

Although we did not always have more than one BP measurement, it is obvious that even though patients had a high-risk profile they were not adequately monitored for BP (22 patients did not have any measurements). It is a severe problem that the BP is not adequately monitored since it is a valid and easy parameter for the daily practical routine helping to assess CVR. The treatment for AI can easily be monitored by measuring the BP and it could help to identify an over or under treatment with GCs as well as with fludrocortisone.

We also found missing attention to weight measurements, as more than 25% (*n* = 83) did not have data for BMI as well as to blood glucose levels, where 76 patients did not have a value for HbA1c (23%).

In comparison to the normal population in Germany, AI patients in this study were more likely to be obese. We found a prevalence of about 1/3, whereas data from 2017 showed a prevalence of 16% obesity in Germany [35]. Obesity is closely related to DM and hyperlipidemia which further increases the CVR [36, 37]. For ¼ of the patients, no BMI was available which is a lot considering the importance of this factor according to the CVR [38, 39].

A lot of the diabetic patients were not taking any medication even though they still had elevated HbA1c levels. Almost ¼ (23.2%) did not have any data for HbA1c demonstrating that the treating physicians did not sufficiently check the adjustable risk factors.

There were differences in the availability of the subgroups of the lipids. Nevertheless, even the total cholesterol, which was the lipid that was measured in most of the patients, was not available for almost 100 patients (30%). As total cholesterol and LDL cholesterol are the most important lipids for CVR [24], it is obvious, that AI patients were treated inadequately. Only 55 of 165 patients with elevated lipids had documented hyperlipidemia, even though our data proved the diagnosis for the other 110 patients. Furthermore, it is well-known today that Lp(a) is an important CVR factor [40]. Only 54 patients (16.5%) were tested for this risk factor, showing insufficient assessment. This problem may partly be explained by the current unavailability of adequate medication to lower Lp(a) levels because statins do not lower them [26].

Serum UA levels were only evaluated in about 50% of the patients. Even in many patients with known hyperuricemia, no UA level was documented. It is interesting, that even though no hyperuricemia was documented, some patients still took uricostatic drugs. In our study, we found a prevalence of 15.3% with hyperuricemia (according to the serum UA) but in the general population, a prevalence of 21% was found [29]. This suggests that even more patients had hyperuricemia in the group without UA measurements (51.7%). Hypertension may be caused by mechanisms including the activation of the renin-angiotensin system, oxidative stress, endothelial inflammation, endothelin-1 activation, and nitrous oxide reduction [29]. Furthermore, it is linked to obesity mainly in a decreased excretion of UA by dysregulation of adipocytes [29].

Limitations of our study include the fact that we only had data from the endocrinological doctors’ offices. Although most affected patients were not adequately treated concerning CVR factors, it may be possible that the general practitioner controlled the CVR factors without drawing any consequences. Nevertheless, endocrinologists need to be aware of the increased CVR and should keep in mind to check whether existing CVR factors have been treated sufficiently.

In addition, checking blood pressure should comprise repeated measurements in a doctor’s office that yield values of 140/90 mmHg or higher [41]. In some cases, we did not have more than one measurement. Furthermore, it was not differentiated if the measurement was done in the doctor’s office or if it was a self-measurement at home.

Finally, as only 40 patients had all data for CVR factors (12.2%), it is conceivable that the endocrinologists did not adequately address the CVR for AI patients. Our data points out a missing awareness of their CVR profile.

We recommend developing a standardized procedure for patients with AI to minimize cardiovascular events and ensure the best possible treatment for these patients. A standardized simplified questionnaire could be used to assess most of the important risk factors, including family history. Nevertheless, treating physicians should be advised to supervise the cardiovascular status regularly and help to reduce the modifiable risk factors. Firstly, regular monitoring of BP, lipid profile, glucose metabolism, and body composition should be integral components of patient care. Early detection of abnormalities allows for timely interventions to mitigate CVR [42]. Secondly, individualized glucocorticoid replacement regimens tailored to physiological requirements and circadian rhythms should be prioritized to minimize metabolic disturbances and optimize cardiovascular health [43, 44]. Thirdly, lifestyle modifications, including dietary adjustments, regular physical activity, smoking cessation, and stress management, should be emphasized to promote cardiovascular well-being [45–47]. Furthermore, judicious use of pharmacological agents, such as statins, antihypertensives, antidiabetic medications, and uricostatic drugs may be warranted to manage specific risk factors [48]. Importantly, interdisciplinary collaboration between endocrinologists, cardiologists, and primary care providers is essential to ensure holistic care delivery and optimal cardiovascular outcomes in this vulnerable population.

In conclusion, our study confirms the high prevalence of CVR factors in AI patients and reveals the serious lack of awareness regarding not only their disease [49, 50] but also their increased CVR profile and the urgent need for medical interventions in order to improve patients’ outcome. Further studies should also focus on the interactions of total daily GC dose, the type of GC treatment, the duration of AI and the presence of CVR.

## Data Availability

No datasets were generated or analysed during the current study.

## Appendix

**Diagnostic reference levels** Table 1**:**

**Table 1:** Diagnostic reference levels

Measurements	Low	Normal	Elevated			
Cholesterol [mg/dl]	≤150	150–240	≥240			
Triglycerides [mg/dl]		≤200	>200			
LDL [mg/dl]		<155	≥155			
HDL [mg/dl]	≤35	>35				
Lipoprotein a [nmol/l]		≤72	>72			
Uric acid [mg/dl]		≤5, 7	>5, 7			
HbA1c [%]		≤5, 7	>5, 7	Prediabetes: 5,7–6,5	Limits for patients with DM: 6, 5–7,5	
BMI [kg/m^2^]		<25	Overweight: ≥25 – <30	Obesity Class I: ≥30 – <35	Obesity Class II: ≥ 35 – <40	Obesity Class III: ≥40
Systolic blood pressure [mmHg]		<140	≥140			
Diastolic blood pressure [mmHg]		<90	≥90			
